# Agricultural Drought Monitoring via the Assimilation of SMAP Soil Moisture Retrievals Into a Global Soil Water Balance Model

**DOI:** 10.3389/fdata.2020.00010

**Published:** 2020-04-09

**Authors:** Iliana E. Mladenova, John D. Bolten, Wade Crow, Nazmus Sazib, Curt Reynolds

**Affiliations:** ^1^NASA GSFC, Hydrological Sciences Lab (617), Greenbelt, MD, United States; ^2^UMD, Earth System Science Interdisciplinary Center, College Park, MD, United States; ^3^USDA ARS, Hydrology and Remote Sensing Lab, Beltsville, MD, United States; ^4^Science Application International Corporation, Lanham, MD, United States; ^5^USDA FAS, Washington, DC, United States

**Keywords:** agricultural drought, soil moisture, SMAP, hydrologic modeling, data assimilation

## Abstract

From an agricultural perspective, drought refers to an unusual deficiency of plant available water in the root-zone of the soil profile. This paper focuses on evaluating the benefit of assimilating soil moisture retrievals from the Soil Moisture Active Passive (SMAP) mission into the USDA-FAS Palmer model for agricultural drought monitoring. This will be done by examining the standardized soil moisture anomaly index. The skill of the SMAP-enhanced Palmer model is assessed over three agricultural regions that have experienced major drought since the launch of SMAP in early 2015: (1) the 2015 drought in California (CA), USA, (2) the 2017 drought in South Africa, and (3) the 2018 mid-winter drought in Australia. During these three events, the SMAP-enhanced Palmer soil moisture estimates (PM+SMAP) are compared against the Climate Hazards group Infrared Precipitation with Stations (CHIRPS) rainfall dataset and Normalized Difference Vegetation Index (NDVI) products. Results demonstrate the benefit of assimilating SMAP and confirm its potential for improving U.S. Department of Agriculture-Foreign Agricultural Service root-zone soil moisture information generated using the Palmer model. In particular, PM+SMAP soil moisture estimates are shown to enhance the spatial variability of Palmer model root-zone soil moisture estimates and adjust the Palmer model drought response to improve its consistency with ancillary CHIRPS precipitation and NDVI information.

## 1. Introduction

Timely and routine knowledge of expected crop yield production is important for farmers, agricultural agencies, insurance companies, aid organization and other end-users because it drives food supply/price projections, impacts trade markets, and identifies food insecure areas. However, yield forecasting using physically-based crop growth models is challenging and requires accurate knowledge of a variety of environmental parameters (Basso et al., 2013; Di Paola et al., 2016). Important inputs for these models include: weather-related data (e.g., precipitation, radiation, and temperature), environmental and crop characteristics (e.g., soil properties, crop type, and crop growth stages), and agricultural and management practices (e.g., crop calendar, irrigation schedule, and fertilization information).

The two factors of the highest significance for crop growth and yield accumulation are heat (i.e., air temperature and radiation budget) and plant available water (i.e., soil moisture) (Lobell et al., 2009). These factors regulate and control all vital physiological and chemical crop processes such as the amount of energy absorbed by the crop, water and nutrient uptake and movement, transpiration, and photosynthesis. However, each crop has its own ideal heat and soil moisture requirements for achieving optimal yield potential, and weather extremes can cause significant deviations from these ideal conditions and result in sub-optimal crop production.

Drought is of great importance to agriculture. It is typically characterized by a precipitation deficiency and high air temperature that results in reduced infiltration, runoff, and ground water recharge and an increase in evaporation/transpiration (Keyantash and Dracup, 2002; Boken et al., 2005). From an agricultural perspective, drought refers to the deficiency of plant-available water in the root zone of the soil profile relative to normal conditions over a persistent period of time (Boken et al., 2005). The inability to replace crop evapotranspiration losses due to insufficient soil water storage causes plant water stress, which, in turn, results in reduced biomass and yield (Keyantash and Dracup, 2002). Thus, root-zone soil moisture (RZSM) is of great interest for operational agriculture monitoring and in-season crop growth modeling.

This paper focusses on the value of a specific RZSM soil moisture data set operationally produced for the U.S. Department of Agriculture-Foreign Agricultural Service (USDA-FAS). Important operational USDA-FAS goals include maintaining global food security and commodities market development. In particular, USDA-FAS's Office of Global Analysis (USDA-FAS/OGA) serves as a major source of objective and reliable global agricultural production information for the USDA's monthly World Agricultural Supply and Demands Estimates (WASDE) report—the primary source of the USDA's global commodity outlook. The monthly WASDE report provides public access to information affecting world food security and is crucial for supporting decisions affecting U.S. agriculture, trade and food aid policies—as well as aiding economic decisions made by stakeholders, policymakers, and governments. USDA-FAS/OGA uses a variety of data products at regional, national and subnational scales to operationally monitor and analyze monthly changes in global crop production. The analysis of RZSM patterns, and their relationship to climatological expectations, is an important part of this analysis. Within the United States, an analogous domestic monitoring role is played by the USDA National Agricultural Statistical Service (USDA NASS).

Prior to 2014, USDA-FAS RZSM information was generated using a modified two-layer Palmer model (PM). The PM is a simple soil moisture accounting model driven by daily meteorological data (including daily precipitation estimates and temperature observations) provided by the U.S. Air Force 557th Weather Wing (USAF). It applies a simple water balance formulation to estimate temporal variations in RZSM. However, this approach shows less skill in the case of poor-quality forcing data. In particular, random errors in daily precipitation forcing propagate through modeled soil water balance physics and can greatly reduce the accuracy of the model-generated soil moisture estimates (Reichle and Koster, 2004, 2005).

Precipitation related errors are effectively filtered via the sequential assimilation of satellite-based soil moisture observations, and numerous studies have demonstrated the ability of land data assimilation systems to enhance the value of the model RZSM estimates for yield forecasting and early agricultural drought detection (de Wit and van Diepen, 2007; Crow et al., 2008; Bolten et al., 2010). Therefore, starting in 2014, the operational USDA-FAS Palmer model was enhanced by adding a data assimilation system capable of ingesting surface soil moisture retrievals acquired from satellite-based passive microwave sensors. The USDA-FAS has adopted the resulting product as a baseline RZSM estimator and incorporated its output into the operational Crop Assessment Data Retrieval and Evaluation (CADRE) Data Base Management System (DBMS) (Bolten et al., 2010; Bolten and Crow, 2012; Crow et al., 2012; Han et al., 2014). To this end, USDA crop analysts use data compiled within the CADRE DBMS, including this satellite-enhanced RZSM and a convergence of evidence methodology, to maximize the accuracy of the USDA-FAS international yield forecasts.

The latest refinement to the satellite-enhanced PM system is based on ingesting near-real time, daily ground soil moisture observations acquired from the NASA Soil Moisture Active Passive Mission (SMAP) mission (Mladenova et al., 2019). The ingestion of SMAP soil moisture products, which began operationally in April 2017, was designed to enhance the USDA-FAS's drought monitoring capability by improving the quality of RZSM soil moisture information provided to the USDA-FAS CADRE DBMS. As such, it represents one of the first truly operational implementations of SMAP soil moisture products for decision support.

Our focus here is to evaluate and quantify the added value contributed by assimilating SMAP for the agricultural drought monitoring capacity of the USDA-FAS CADRE DBMS. This will be done by: (1) assessing the impact of SMAP on the USDA-FAS's soil moisture estimates and (2) examining the change in skill (if any) of the SMAP-enhanced PM model (PM+SMAP) for capturing the extent, duration and severity of agricultural drought events. To this end, the performance of the USDA-FAS's soil moisture information (without and with SMAP) will be examined for its capability to detect RZSM deficiency over three agricultural regions that have experienced major agricultural drought since the launch of SMAP in early 2015: (1) the 2015 drought in California (CA), USA, (2) the 2017 drought in South Africa, and (3) the 2018 mid-winter drought in Australia. RZSM deficiency will be established by evaluating the behavior and accuracy of the model standardized soil moisture anomalies in relation to change in anomalies in precipitation and the Normalized Difference Vegetation Index (NDVI).

Therefore, this paper is organized as follows. Section II provides background on the physics of agricultural drought and its impact on crop growth and yield production—as well as describing available soil moisture-based indices with demonstrated value for agricultural drought monitoring. Section III focuses on the soil moisture, precipitation, and NDVI datasets and provides a description of the study domains and the drought events listed above. Results will be presented/discussed in section IV and summarized in section V.

## 2. Background: Agricultural Drought and Soil Moisture Indices

Agricultural drought is generally associated with a decline in precipitation that causes insufficient plant water availability and reduced soil water storage. Its adverse effect can be accelerated by the simultaneous occurrence of high air temperatures and strong winds, which causes further drying. Boken et al. (2005) describe agricultural drought as “the most complex category of drought” since it cannot be measured immediately due to the delay between its manifestation and the eventual determination of its magnitude (via yield measurements made at the end of the growing season). In addition, the relationship among plants, weather, and environmental conditions is not linear. Therefore, below-average precipitation does not cause an immediate decline in crop health—as plants have several short-term adaptive mechanisms to deal with a brief shortage of water and/or heat stress (Slayter, 1967; Jensen, 1968; Wani et al., 2009). In relation to plant development and health and yield formation, the three most important aspects of agricultural drought are its duration (i.e., how long is the period of limited soil moisture conditions), timing (i.e., when does the water stress occur during the growing season), and severity (Abendroth et al., 2011; Licht, 2014; Mladenova et al., 2017). Plant water requirements vary strongly during the growing season. Crop yields are generally most susceptible to water deficiency during the reproductive crop development stage associated with grain-filling and formation. As these requirements are relatively well-known, the eventual (yield-based) impact of drought can often be estimated within season.

A large number of indices have been developed to monitor drought and quantify its impact. Mishra and Singh (2010) offer a comprehensive overview of commonly used drought indices and a detailed discussion of their advantages and disadvantages. Overall, each index explores a different component of the water cycle (i.e., precipitation, runoff, groundwater storage, and evapotranspiration) or environmental variable (i.e., land surface temperature and NDVI) and its relation to drought.

Here our primary focus will be on soil moisture-based indices. Narasimhan and Srinivasan (2005) proposed a Soil Moisture Deficit Index (SMDI) based on simulated soil moisture values acquired from the SWAT (Soil Water Assessment Tool). SMDI monitors drought by scaling the most recent weekly soil moisture conditions relative to long-term median, minimum, and maximum expectations. Several authors have proposed soil moisture indices where drought conditions are assessed based on rescaling estimated soil moisture content relative to soil-specific properties such as available water content (AWC), field capacity (FC), or wilting point (WP) (Sridhar et al., 2008; Hunt et al., 2009; Martínez-Fernández et al., 2015). Zhou et al. (2017) advanced this approach by applying a root-weighting function, where a soil moisture index is corrected for crop rooting depth and root-length density. SMDI requires that the inter-annual and seasonal effects are properly accounted for, which, in turn, requires an adequate long-term climatology. The soil property rescaled indices, on the other hand, require reliable AWC, FC, and WP, and results can vary widely based on the approach used to obtain these parameters (Martínez-Fernández et al., 2016). In addition, the actual rooting depth and soil volume from which plants extract water changes throughout the growing season. As a result, applying this approach at a global scale is challenging.

Another alternative is the calculation of standardized anomaly indices that express current soil moisture conditions relative to a climatological expectation. This can be useful to convey degree and duration of drought across several climatological regions and multiple soil and vegetation types over time. If standardized by a long-term standard deviation, such an index represents standardized anomalies that summarizes the deviation of the current soil moisture conditions in terms of standard deviations relative to long-term climatological expectations for a given month. The monthly Standardized Anomalies (*SA*) used here is computed as:

(1) $$\begin{array}{l} SA_{i}=\left(RZSM_{i}-\mu_{m}\right)/\sigma_{m} \end{array}$$

where *RZSM* is the root-zone soil moisture, *m* represents the climatological value sampled for a particular month of the year, *i* indicates the monthly time series values, and μ and σ indicate mean and standard deviation *SM* values for the specific month *m*. For example, the *SA* for April 2013 is estimated by calculating μ and σ utilizing soil moisture observations sampled from all Aprils in the historical data record.

Here, the *SA* approach is applied because it is easily applicable at a global scale and does not require any additional ancillary data inputs. In addition, it offers the opportunity to compare different components of the hydrologic cycle (e.g., soil moisture vs. precipitation) and measurements reflecting vegetation status (e.g., NDVI). Since the RZSM product is an important part of the official product suite currently generated for USDA-FAS, an anomaly analysis carried out as described above should provide useful insight into the utility of this product for the USDA-FAS crop analysts. Note that the interpretation of (1) is typically based on an implicit assumption of a Gaussian RZSM distribution. More robust non-parametric percentile or return interval approaches are also possible but require longer sampling periods than the relatively short data records provided by existing global RZSM products (see below).

## 3. Approach

### 3.1. Soil Water Balance Modeling

As discussed above, the USDA-FAS soil moisture product is based on the modified 2-layer Palmer model (PM) (Palmer, 1965; Bolten et al., 2010; Bolten and Crow, 2012). The top layer is assumed to have a maximum water holding capacity of 25.4 mm, while the amount of the water that can be stored in the root-zone is modeled as a function of specific soil properties. The PM is a simple 2-layer bucket-type of soil water balance model driven by daily observations of precipitation and minimum and maximum air temperature. The USAF precipitation data used to force the model is generated as a part of the agency's Agricultural Meteorology modeling system. The precipitation estimates are acquired in near-real-time by merging observations from the Special Sensor Microwave/Imager (SSM/I) satellite, geostationary satellites such as Geostationary Operational Environmental Satellite (GOES) and Meteosat, and rain gauge data from the World Meteorological Organization (WMO). Here, the PM was applied daily at a global scale on a regular 0.25° grid to calculate daily RZSM products via its soil water balance formulation.

### 3.2. SMAP Level 2 Soil Moisture

The SMAP satellite was launched in January of 2015 and began providing scientific data on March 31, 2019. It is an L-band mission that observes the Earth's surface at about 40-km resolution twice a day at 6 a.m. and 6 p.m. (local solar time). SMAP was designed to carry aboard two microwave instruments, a radar (centered at 1.26 GHz) and a radiometer (centered at 1.5 GHz). Unfortunately, the SMAP radar failed in July 2015. However, the passive microwave radiometer has continued to generate a nearly continuous data record since late March 2015.

SMAP generates several different global soil moisture products using various radiative transfer-based algorithms and methodologies. Of particular interest here is the Level-2 passive-based only soil moisture product (L2_SM_P) (ONeill, 2018). The L2_SM_P baseline soil moisture is retrieved using V-pol brightness temperature data and the so-called “Single Channel Algorithm” (Jackson, 1993; Chan, 2016). This data product is distributed at a 36-km × 36-km EASE2 grid projection. Prior to assimilation into the PM model (see below), L2_SM_P retrievals were re-projected and re-sampled to match the 0.25° PM grid using tools developed by the SMAP science team. The SMAP L2_SM_P product has been extensively validated over a large number of validation sides and using ground data collected during a number of field campaigns specifically designed to support the SMAP Calibration/Validation (Cal/Val) activities (Chan, 2016; Chan et al., 2016; Burgin et al., 2017; Cai et al., 2017; Colliander et al., 2017).

### 3.3. Data Assimilation

The current operational USDA-FAS SMAP-based RZSM product is derived based on the assimilation of the SMAP L2_SM_P soil moisture product (section 3.1) into the 2-layer PM (section 3.1) to filter random errors that degrade PM RZSM estimates. This assimilation is based on an Ensemble Kalman filtering (EnKF) approach. The EnKF, its background and application in hydrology—as well as its specific USDA-FAS implementation—have been well-documented by previous studies (Reichle and Koster, 2004, 2005; Bolten et al., 2010; Bolten and Crow, 2012; Han et al., 2014). In particular, the specific data assimilation system applied to generate the existing USDA-FAS RZSM products is described in detail by Mladenova et al. (2019). Therefore, only a few key elements relevant to the specific USDA-FAS SMAP implementation are summarized here.

The EnKF is a sequential data assimilation technique where the update of the model forecasts in response to the acquisition of uncertainty observations is based on error covariance information sampled from a Monte Carlo model forecast ensemble. Here, this ensemble is generated by applying random noise directly to 2-Layer PM states. As a result, the EnKF is indirectly driven by two user-defined parameters: the forecast error noise covariance matrix *Q* (used to drive the generation of the Monte-Carlo PM ensemble) and the observation covariance matrix *R* (which reflects our degree of confidence in the accuracy of assimilated observations). *Q* and *R* are generally assumed constant in time here—see Mladenova et al. (2019).

Uncertainty in the PM model forecasting represents a primary source of the model forecast uncertainty represented by *Q*. As noted above, USAF precipitation forcing estimates are generated by merging various sources including the World Meteorological Organization (WMO) ground stations. Given the general understating that gauge-based correction is important for ensuring the quality of precipitation forcing data, *Q* was modeled as an increasing function of the distance between the model grid pixel and the nearest gauge station—thereby lending more confidence to background PM forecasts over areas with greater station density coverage (Bolten and Crow, 2012).

Similar ground-based quality concepts were applied when parametrizing *R*. Generally, the performance of the passive-based soil moisture retrieval algorithms and the quality of the corresponding estimates are highly dependent on the vegetation layer. Canopy density and the amount of water present in the plants both tend to prevent the microwave signal from fully penetrating through the vegetative canopy. Even though the SMAP baseline retrieval algorithm implements a vegetation correction step, canopy attenuation still causes higher soil moisture retrieval uncertainty over densely vegetated areas. The SMAP Calibration and Validation (Cal/Val) team has evaluated SMAP soil moisture retrievals using very comprehensive and well-calibrated ground-collected datasets (Colliander et al., 2017). Using this information and algorithm sensitivity analysis, they have developed uncertainly flags that allow the user to mask out unreliable retrievals (including retrievals over densely vegetated areas). In addition, the Cal/Val team generates regular error statistics computed against *in situ* data as a function of land cover type. Therefore, *R* values used here were based on the unbiased component of the Root Mean Squared Error (*ubRMSE*) values provided by the SMAP Cal/Val team for specific vegetation classes (Jackson et al., 2016).

### 3.4. Evaluation Data

Given the overall lack of ground-based RZSM observations, and the difficulty of scaling available observations up to a coarse-scale average, we will employ alternative approaches for assessing value in our data assimilation results. However, it should be noted that more traditional verification of the USDA-FAS SMAP-based RZSM product against ground-based soil moisture is described in Mladenova et al. (2019).

Globally, most agriculture production is still rain-fed. Thus, temporal and spatial variations in precipitation play an important role in crop growth. With this in mind, we can leverage the relationship between root zone soil moisture dynamics and precipitation and vegetation dynamics in regions of rain-fed agriculture. Since the PM is governed primarily by precipitation forcing, PM soil moisture patterns reflect patterns in the precipitation forcing used by the PM. However, the assimilation of SMAP into the PM (hereinafter referred to as the PM+SMAP case) should allow for the representation of more realistic soil moisture patterns than the baseline USAF-forced PM. Therefore, one technique for demonstrating the value of a data assimilation-based system combining both satellite-based precipitation and soil moisture observations is to carefully analyze the spatial and temporal agreements between the datasets (i.e., PM RZSM and PM+SMAP results), as well as the datasets used in their production, and compare them to a higher-accuracy precipitation dataset (i.e., the consolidated CHIRPS data set). This approach is followed here. Specifically, we compare both the PM and PM+SMAP results with the independent CHIRPS precipitation dataset.

An additional comparison analysis against NDVI anomalies is conducted to evaluate the impact of water shortage on vegetation health as captured by NDVI. Is it well-known that, in mid-latitude agricultural regions during the growing seasonal, negative anomalies in RZSM tend to produce a time-lagged corresponding negative anomaly in NDVI (Adegoke and Carleton, 2002). The strength of association between RZSM and NDVI have been used to assess the value of the PM+SMAP case under the assumption that—when comparing multiple products in a water-limited environment—the highest-quality RZSM product should also possess the largest lagged correlation with independent NDVI estimates of biomass and vegetation health (Bolten and Crow, 2012; Crow et al., 2012; Han et al., 2014). However, as discussed below, uncertainty regarding the exact temporal relationship between RZSM and NDVI anomalies can complicate this interpretation.

Based on this two-tiered approach, we aim to determine if applying SMAP-based soil moisture in an EnKF framework leads to more realistic patterns of wetness and vegetation dynamics by comparing them to satellite-based CHIRPS precipitation and MODIS-based NDVI.

#### 3.4.1. CHIRPS Precipitation

As described above, we applied a data denial validation precipitation data to assess the ability of SMAP data assimilation to compensate for random precipitation forcing errors. Hence, baseline RZSM results were based on forcing the PM using the Climate Hazards group Infrared Precipitation with Stations (CHIRPS) dataset. CHIRPS is distributed at 0.05° and offers a quasi-global coverage (50°S-50°N). CHIRPS incorporate global climatologies, several different satellite-based products and *in situ* station data. Thus, the CHIRPS algorithm encompasses three major components: (a) the Climate Hazards group Precipitation climatology (CHPclim), (b) the satellite-only Climate Hazards group Infrared Precipitation (CHIRP), and (c) the station blending procedure that produces the CHIRPS. The global monthly climatologies are based on station data obtained from the Agromet Group of the Food and Agriculture Organization of the United Nations (FAO) and the Global Historical Climate Network (GHCN). In addition to the gauge data, CHPclim incorporates elevation, latitude and longitude information as well as monthly long-term mean values acquired from Tropical Rainfall Measuring Mission 2B31 microwave precipitation estimates, CMORPH microwave-plus-infrared based precipitation estimates, monthly mean geostationary infrared brightness temperatures and land surface temperature estimates. CHPclim uses a moving window method to build tile specific regression models that are consequently fit to the FAO climatology. Station specific biases are then computed against the GHCN data. Lastly, CHIRP is blended with station observations from several public regional archives. Merging is done by computing bias ratios between the CHIRP data and the nearest 5 stations. These bias ratios are used to compute a correction factor that is used to adjust the original CHIRP values. Final CHIRPS estimates are a weighted combination of the original CHIRP and the adjusted CHIRP, where the weighing function is determined based on the agreement of each product with the nearest station. A detailed description of the CHRIP/S methodology and validation results can be found in Funk et al. (2015), Paredes-Trejo et al. (2017), and Rivera et al. (2018).

CHIRPS precipitation is an independent data set as it was not used to run the PM and is considered to be of relatively high-quality (vs. the USAF precipitation product used to drive the PM) due to its more complex blending algorithms, the use of global precipitation climatology and the much larger number of gauge data incorporated into the CHIRPS product, as discussed above. However, as mentioned, it a quasi-global data set and does not provide full global coverage (50°S-50°N). In addition, there is considerable latency in data availability (about 2 days for the preliminary product and about 3 weeks for the final CHIRPS data used here; see Funk et al., 2015). Therefore, unlike the USAF precipitation product used operationally by USDA-FAS to generate PM RZSM estimates, the final CHIRPS product is not appropriate for near-real time operational implementation.

Preliminary analyses (not included in this paper) show that the CHIRPS temporal and spatial response is consistent with other precipitation data sets such as the Parameter-elevation Relationships on Independent Slopes Model (PRISM) and the South African Weather Service data. However, both of these products have only regional coverage (i.e., the contiguous United States and South Africa, respectively) and cannot support a global analysis.

#### 3.4.2. GIMMS MODIS NDVI

NDVI anomalies were used here as a proxy of crop status and vegetation health. The Moderate Resolution Imaging Spectroradiometer (MODIS) NDVI data used in this study was processed by the NASA/Goddard Space Flight Center's Global Inventory Modeling and Mapping Studies (GIMMS) Group through funding support of the Global Agricultural Monitoring project by USDA-FAS. It is generated using the maximum-value compositing technique (Tucker et al., 2005; Brown et al., 2006). As discussed above, NDVI is used to evaluate the ability of various RZSM products to predict inter-annual variations in crop status and/or vegetation health.

The standardization procedure described in (1) is applied to develop monthly standardized precipitation and NDVI standardized anomalies. A “^*^” superscript is used to indicate standardized anomalies (SA). Therefore, PM^*^ is RZSM SA of the Palmer model alone and ENKF^*^ refers to SA produced via the assimilation of SMAP into the PM (PM+SMAP). Likewise, CHIRPS^*^ and NDVI^*^ refer to monthly SA anomalies acquired from the CHIRPS precipitation and the GIMSS NDVI products, respectively.

### 3.5. Domains Description

**Figure 1:**
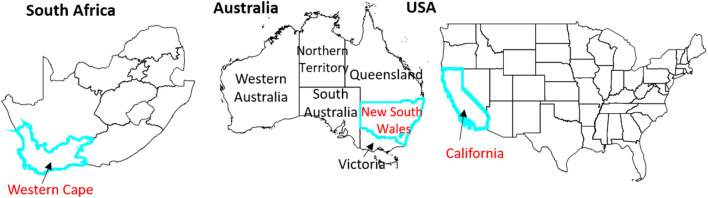
Study areas.

As mentioned above, our three study domains of interest are: California (USA), the Western Cape (South Africa) and New South Wales (Australia) (Figure 1). Each of these regions experienced at least one major agricultural drought event within the SMAP data era (i.e., late March 2015 to present).

Along with the WASDE report, USDA-FAS routinely releases Commodity Intelligence Reports (CIR), and World Agricultural Production (WAP) Circulars. These briefs describe changes in crop status and expected yield variations for a specific country or agricultural area in the context of an ongoing weather or climate event. The impact of the 2017 drought in South Africa and the 2018 mid-winter drought in Australia were extensively described by the USDA-FAS briefs. Likewise, the Californian drought is described by USDA NASS reports. These reports are referenced below to provide context concerning the impact of our three selected drought events on large-scale agricultural productivity.

#### 3.5.1. Western Cape, South Africa (2017)

Our first case study focuses on the impact of a prolonged drought in the Western Cape province of South Africa. The Western Cape is located in the south-western edge of South Africa. It is the country's largest wheat producing region and operates on an austral winter wheat calendar with planting in May and harvest in November. The agriculture in the area is predominantly rain-fed. Climate in the Western Cape is characterized by hot/dry summers and cold/wet winters. Average annual rainfall is 185 mm, which is significantly lower than the South African national average of 380 mm (statistics are based on the 1981–2018 CHIRPS data record). The Western Cape Province has experienced below-average rainfall since 2014/15—reaching near-record lows during the 2017 growing season, which was the lowest recorded since either 1981 (based on CHIRPS data record) or 1933 (based on the South African Weather Service stations; see Wolski, 2018). As result, the 2017 growing season was categorized as the worst since 1904 (Botai et al., 2017; Wolski, 2018). In February of 2018, the South African Crop Estimating Committee predicted that the 2017/2018 wheat yield would fall 64% below its 5-year average (see USDA-FAS Commodity Intelligence Report from February 8, 2018; https://ipad.fas.usda.gov/highlights/2018/02/SouthAfrica/index.pdf).

#### 3.5.2. New South Wales, Australia (2018)

The New South Wales (NSW) province, located in the south-eastern corner of Australia, is one of the major agricultural production areas in Australia. Its climate varies from arid to semi-arid, in areas to the west of the Great Dividing Range, to more oceanic and humid subtropical in the east. Average annual rainfall is about 365 mm (based on the 1981–2018 CHIRPS data record). Wheat, canola and barley are the major commodities cultivated in the area. Winter crops are planted between May and July and harvested between late October and early January. However, specific planting dates are highly dependent on winter rainfall amounts. Numerous studies have found that Australia is prone to inter-annual drought due the impact of several major climate phenomenon such as El Niño/Southern Oscillation (ENSO), Pacific Decadal Oscillation (PDO), and the Indian Ocean Dipole (IOD) (Ummenhofer et al., 2009; Verdon-Kidd and Kiem, 2009; van Dijk et al., 2013). For example, the 2018 rainfall season brought below-average rainfall causing delay in sowing operations and postponed planting. USDA-FAS winter wheat crop statistics for the 2018 winter season are given in Table 1. The corresponding reduction in expected yields for 2018 was attributed to a persistent deficit of RZSM caused by the below-average precipitation starting from the beginning of the austral winter growing season (i.e., in and around May 2018).

**Table 1:** USDA-FAS 2018 Winter Wheat Monthly Crop Statistics.

**Report date**	**Crop**	**Harvested area**	**Expected yield**
July 2018	↑3%	↓2%	↑5%
September 2018	↓6%	↓10%	Same as last year
October 2018	↓13%	↓10%	18% below the 5-year average
November 2018	↓18%	↓12%	21% below the 5-year average
December 2018	↓20%	↓18%	17% below the 5-year average

*↑ and ↓ indicate % up and down from last year. The specific CIR and WAP Circular Series can be found at https://ipad.fas.usda.gov/search.aspx*.

#### 3.5.3. California, USA (2015)

**Figure 2:**
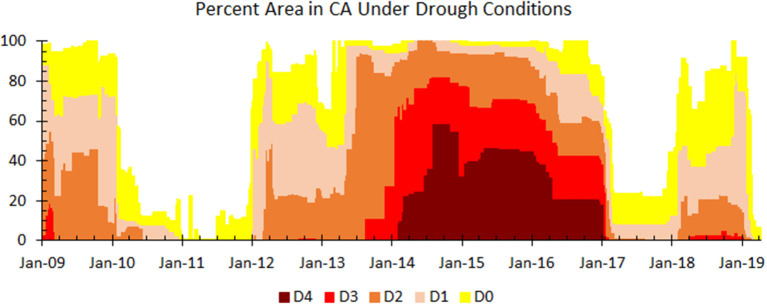
According to the United States Drought Monitor, percent area of CA, USA facing drought conditions between 2009 and (early) 2019. D4 Exceptional Drought, D3 Extreme Drought, D2 Severe Drought, D1 Moderate Drought, and D0 Abnormally Dry Data Source: US Drought Monitor (https://droughtmonitor.unl.edu/Data.aspx).

California (CA) typically receives most of its annual precipitation during the winter season (November through April). However, the 2013/2014 winter season was the 6th driest since 1985 (Seager et al., 2015). Furthermore, the 2011–2014 3-year winter average precipitation and air temperature were the 2nd lowest and the warmest, respectively, on record since 1985 (Vose et al., 2014; Seager et al., 2015). Anomalously high temperatures intensified a RZSM deficit by increasing the amount of soil water lost to evapotranspiration. According to the US Drought Monitor (https://droughtmonitor.unl.edu/), as of early April 2015, 44% of CA was experiencing exceptional drought conditions (D4 on the Drought Monitor severity scale). This percentage increased to almost 47% by mid-May (Figure 2). Thus, 2015 was the fourth year of profound multi-year drought event.

CA is one of the major agricultural areas in the US. Commodities grown in the area are diverse and include a large variety of fruits, nuts and vegetables—as well as additional field crops (https://www.nass.usda.gov/Quick_Stats/Ag_Overview/stateOverview.php?state=CALIFORNIA). An important characteristic of CA agriculture is its heavy reliance on irrigation, which supports the growth of high-value cash value crops such as nuts and vegetables. As a result, the state of CA has the largest number of acres of irrigated farmed land in the U.S. (Johnson and Cody, 2015). According to the California Department of Water Resources, about 41% of total water withdrawals in the state are allocated for irrigated agriculture (based on 2010 data). Therefore, plant growth is not solely dependent on precipitation and inter-annual differences in crop productivity are often buffered. For example, during 2009–2012 (a low drought stress period) and 2014–2017 (a high drought stress period) 4-year almond yield averaged were nearly identical (i.e., 2,215 and 2,105 lb/acre, respectively based on the USDA-NASS survey program, https://quickstats.nass.usda.gov/). This lack of inter-annual variability highlights the importance of irrigation in CA and its ability to moderate drought impacts on plant growth and lower agricultural crop productivity.

## 4. Results

**Figure 3:**
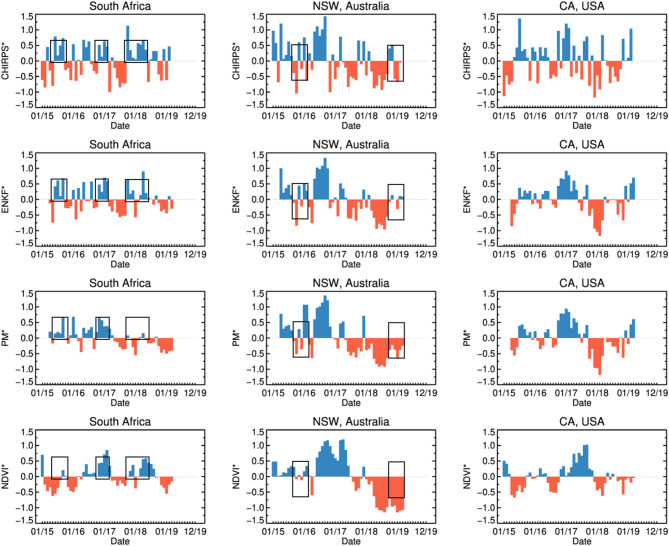
Time series of monthly standardized RZSM anomalies (ENKF* and PM*), precipitation anomalies (CHIRPS*), and vegetation status anomalies (NDVI*) for the Western Cape, South Africa (column 1), NSW, Australia (column 2), and CA, USA (column 3) domains shown in Figure 1. Black boxes highlight periods of time where SMAP assimilation had a notable impact on RZSM* results. Values represent domain averages.

**Figure 4:**
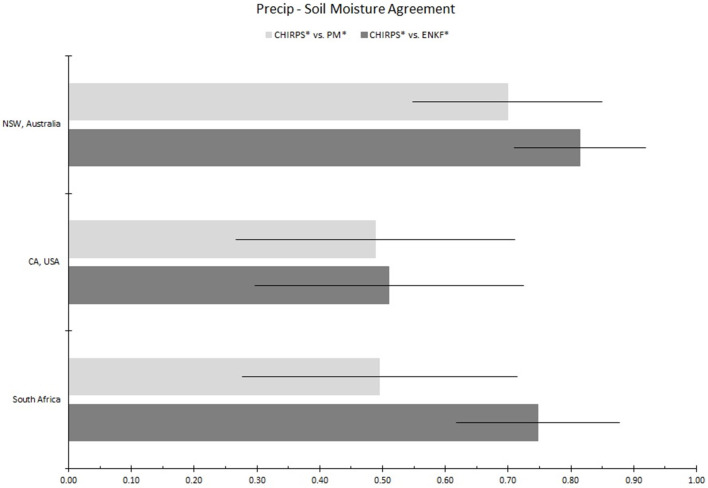
Temporal agreement between monthly standardized RZSM anomalies (ENKF* and PM*) and precipitation anomalies (CHIRPS*) over all three study regions. Dark gray and light gray colored bars show sampled correlation between CHIRPS* and ENKF* and CHIRPS* and ENKF*, respectively. Error bars reflect 95% confidence intervals for these sampled correlations. *Z*-test statistics at α = 0.10 indicated that the *R* values computed with and without SMAP over NSW and South Africa are significantly different.

Our analysis focuses on exploring the agreement between PM RZSM products (acquired both with and without SMAP data assimilation) with independent estimates of rainfall and vegetation health captured by CHIRPS and GIMSS MODIS NDVI, respectively. For all three locations over the entire SMAP data record (March 31, 2015 onward), time series of the standardized anomalies of PM-only RZSM (PM^*^), EnKF-based RZSM (ENKF^*^), precipitation (CHIRPS^*^), and vegetation health (NDVI^*^) standardized anomalies are shown in Figure 3. In addition, temporal correlations between CHIRPS^*^ and both PM^*^ and ENKF^*^ RZSM time series are summarized in Figure 4.

**Figure 5:**
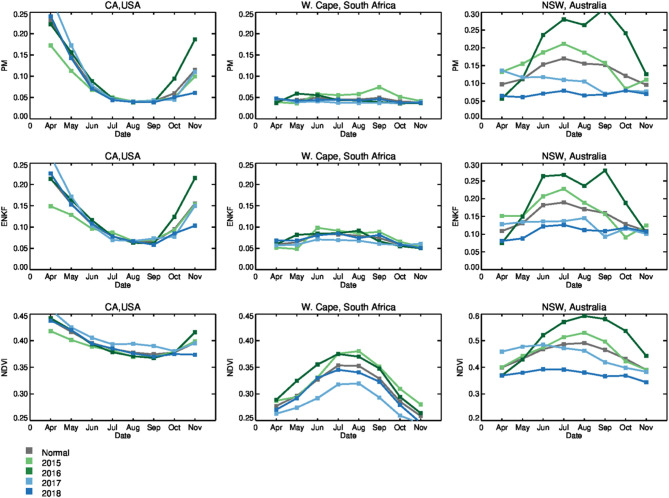
Monthly change in actual soil moisture [m^3^/m^3^] and NDVI [–] over the growing season over 2015–2018 time period. Gray line represents the normal (average) conditions, while the green and blue colored lines show the annual deviation of these variables relative to the normal. Values represent domain averages. The plots for the Western Cape, South Africa and NSW, Australia reflect the winter crop growing season as specified in the USDA-FAS CIR and WAP briefs cited in this paper.

The convergence of evidence approach employed by the USDA-FAS crop analysts is based on evaluating current crop status relative to some expected normal level using a variety of geospatial variables linked to agricultural productivity (e.g., precipitation, air temperature, RZSM, and NDVI). As shown in Table 1, USDA-FAS crop statistics and yield forecasts are reported relative to either the previous year or the most-recent 5-year average. Figure 5 shows monthly changes in RZSM and NDVI during the 2015–2018 growing season. The gray line represents normal conditions, while the green/blue colored lines show the inter-annual deviation of these variables for a specific year relative to normal. A comparable analysis, performed in near-real-time, can be found on the USDA-FAS Crop Explorer web portal (https://ipad.fas.usda.gov/cropexplorer/Default.aspx).

**Figure 6:**
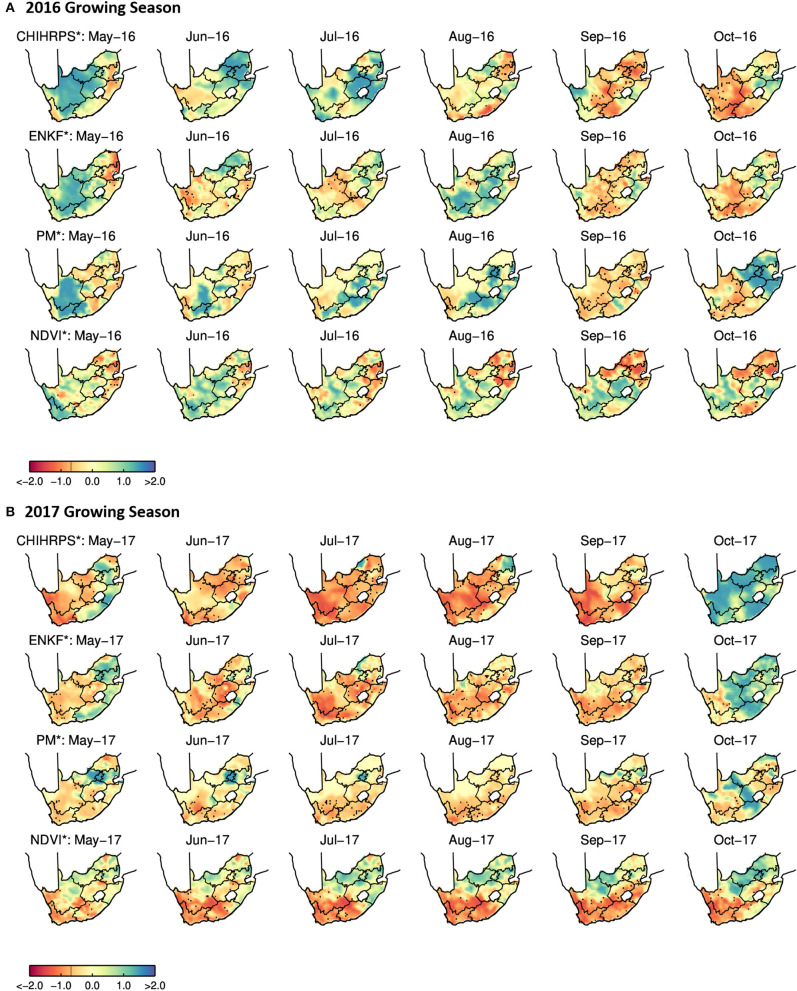
Monthly PM*/ENKF* RZSM, CHIRPS*, and NDVI* anomalies over the Western Cape in South Africa for the 2016 **(A)** and 2017 **(B)** austral winter crop growing season.

**Figure 7:**
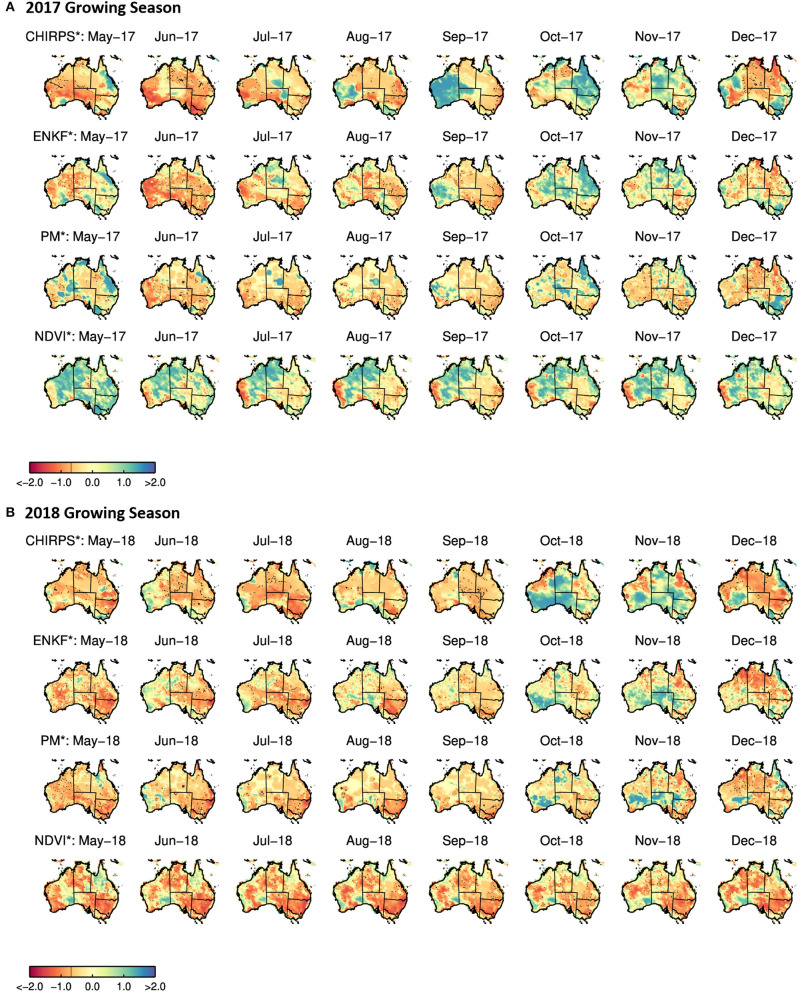
Monthly PM*/ENKF* RZSM, CHIRPS*, and NDVI* anomalies over Australia for the 2017 **(A)** and 2018 **(B)** austral winter crop growing season.

**Figure 8:**
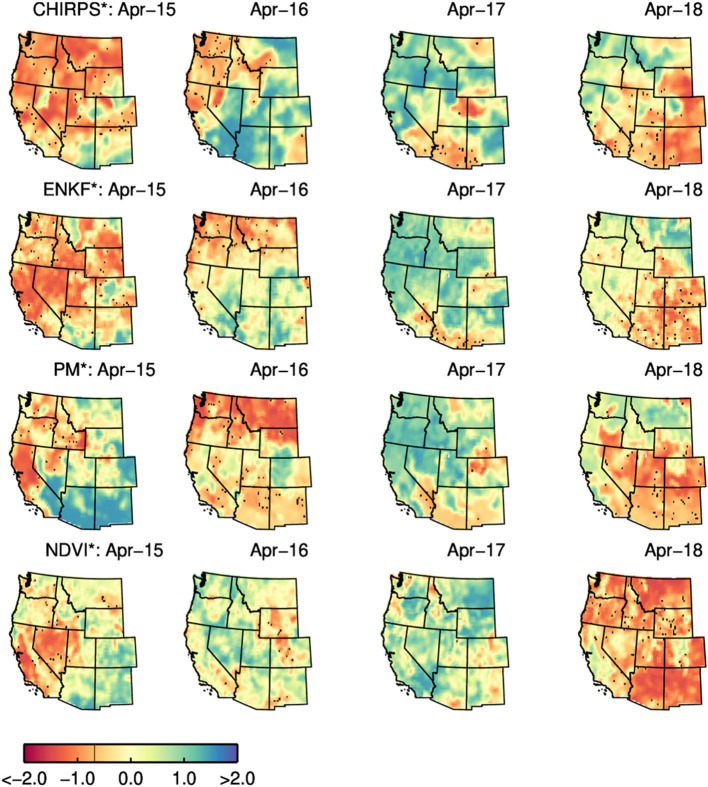
Monthly PM*/ENKF* RZSM, CHIRPS*, and NDVI* anomalies over the western United States for the month of April for the 2015–2018 period.

Note that the plots for the Western Cape and NSW reflect the austral winter crop growing season (roughly November to May). Spatial variability in ENKF^*^ RZSM, PM^*^ RZSM, CHIRP^*^ precipitation, and NDVI^*^ standardized anomalies over South Africa, Australia and the western half of the United States are shown in Figures 6–**8**. Specific results for each of our three case study events are summarized in the next three sub-sections.

### 4.1. Western Cape, South Africa

Time series of the Western Cape ENKF^*^ RZSM, PM^*^ RZSM, CHIRPS^*^ precipitation, and NDVI^*^ in Figure 3 indicate generally good agreement between both RZSM standardized anomaly products and between each RZSM product and precipitation (CHIRPS^*^). However, assimilation of SMAP data (used to generate ENKF^*^ results) introduces additional temporal variability not captured by the original model-only PM^*^ product—see the black outlined boxes in Figure 3. This added variability is in close agreement with observed temporal variations in CHIRPS rainfall and results in the ENKF^*^ product demonstrating enhanced temporal correlation, as compared to model-only PM^*^ results, vs. the CHIRPS^*^ precipitation baseline (see Figure 4).

The impact of SMAP data assimilation is also evident in Figure 5 where PM^*^ results reflect very dry soil moisture conditions and negligible inter-annual variability. The SMAP-based ENKF^*^ results for 2016 and 2018 both show additional information resulting in a slight seasonal peak that is more consistent with the NDVI data. 2017 is the driest year since 1981 (based on CHIRPS data record) in terms of soil moisture over the Western Cape, South Africa (Figure 5 cyan colored line displays the results for 2017).

Spatial variability in RZSM (PM^*^ and ENKF^*^), precipitation (CHIRPS^*^), and NDVI^*^ anomalies over South Africa for two growing seasons (2016 and 2017) are shown in Figure 6. All data sets accurately identify the 2017 growing season as the drier of the two. Drought conditions reach their peak between July and September. Nevertheless, PM^*^ and ENKF^*^ results show different spatial patterns (in e.g., October 2016, May-July 2017, and October 2017). In particular, ENKF^*^ more closely adheres to spatial variability displayed in CHIRPS anomalies—suggesting that the assimilation of SMAP observations is correcting for spatially erroneous rainfall information used to generate the model-only PM^*^ product. This is especially clear in the May-July 2017 imagery where PM^*^ results contain an obvious spatial artifact (indicated by the bright blue circular pattern in Figure 6) in northern South Africa. This artifact originates in the USAF precipitation data and propagates into PM-based RZSM soil moisture estimates. However, the assimilation of SMAP soil moisture retrievals properly filters such spurious patterns and recovers spatial information that is comparable with the spatial patterns captured in the CHIRPS precipitation data.

NDVI also shows that vegetation conditions worsen during the 2017 growing season. However, the spatial pattern of NDVI^*^ does not correlate perfectly with comparable CHIRP^*^ and NDVI^*^ patterns. This is most likely due to a temporal lag in response between the change in plant status and the change in water supply—which can complicate the use of RZSM/NDVI anomaly correlation as an evaluation metric for RZSM time series.

### 4.2. New South Wales, Australia

RZSM^*^, NDVI^*^, and CHIRPS^*^ time series within New South Wales, Australia (NSW; see Figure 3) show good agreement between the RZSM^*^ products and between each RZSM^*^ product and CHIRPS^*^ precipitation. Nevertheless, black boxes in Figure 3 (second column), highlight examples where the ENKF^*^ (generated via the assimilation of SMAP soil moisture products) corrects model-only PM^*^ results in a way that better reflect spatial CHIRPS^*^ patterns. As in the Southern African case, the total temporal agreement between ENKF^*^ and CHIRPS^*^ is higher than that sampled between PM^*^ and CHIRP^*^ (Figure 4)—indicating that SMAP data assimilation is successfully filtering the impact of precipitation errors in PM^*^ results. However, it should be noted that the correlation improvement seen in Figure 4 is relatively small.

Based on the seasonal change in RZSM and NDVI, 2018 can clearly be identified as the worst year in the 2015–2018 SMAP data record for RZSM and vegetation status in the NSW (see Figure 5—the blue line displays results for 2018). In addition, Figure 5 show large interannual variability, which is slightly reduced by the assimilation of SMAP soil moisture (narrower blue error bars, which represent the monthly standard deviation for the ENKF product in the 2015–2018 time period). Figure 7 shows the spatial variability in PM^*^, ENKF^*^, NDVI^*^, and CHIRPS^*^ anomaly patterns over Australia. Overall, the assimilation of SMAP soil moisture improves the spatial coherence between the modeled-based RZSM and CHIRPS precipitation anomalies. Furthermore, the decline in RZSM over the NSW region during the 2018 austral winter growing is clearly captured by both the PM^*^ and ENKF^*^ products—as well as in the precipitation anomalies. NDVI^*^ results show a generally similar trend.

The NSW received badly needed rainfall in October-November 2018. However, the impact of this rainfall is not reflected in an observed increase in NDVI. This is expected since this period of rainfall occurred late in the growing season and coincided with the harvesting period for winter wheat. In addition, during the 2016 growing season, there is a disagreement between the vegetation conditions and precipitation. 2017 begins with above-average rainfall. For example, the Australian Government Bureau of Meteorology reported that the precipitation for the months of January-April 2017 was at or above average and dropped substantially below average only during the month of May (http://www.bom.gov.au/climate/current/statement_archives.shtml). This agrees with the CHIRPS precipitation. Thus, the relatively good vegetation status in May 2017 (see Figure 7) is most likely due to adequate water availability prior to the start of the growing season. As such, it demonstrates the temporal lag discussed above between changes in moisture conditions and its potential delayed impact on vegetation status. This is also confirmed by the decline in vegetation status as a result of the decline in RZSM over the agricultural areas observed later in the growing season (i.e., Victoria, along the coast in South Australia, the eastern half of NSW, the south-eastern corner of Queensland, and the south-western corner of Western Australia).

### 4.3. California, USA

Relative to the South African and Australian results discussed above, comparisons between PM^*^ and ENKF^*^ RZSM time series reveal only a minor impact of SMAP data assimilation on RZSM^*^ in CA (Figure 3). This is almost certainly due to the relatively higher quality of the USAF precipitation forcing data over the United States and our approach of parameterizing the model error—and thus the impact of SMAP soil moisture assimilation—in rain-data-rich areas like the United States. Based on the monthly change in actual soil moisture and NDVI over the CA growing season (Figure 5), PM, ENKF, and NDVI results all show similar seasonal behavior and minimal interannual variability In addition, no discernible SMAP data assimilation impact emerges (that is, no difference between PM and ENKF results). As mentioned above, most of the agriculture in CA is irrigated, which may explain the difficulty of isolating drought events in NDVI^*^ and ENKF^*^ time series results. Nevertheless, 2015 was the 4th year of multi-year drought period. Precipitation amounts during the preceding winter season were much below average. Early on during the 2015 growing season (April-May) soil moisture and vegetation conditions were below normal (Figure 5 light green colored line displays the results for 2015). However, as the growing season develops, irrigation supported by the extraction of groundwater causes RZSM and NDVI values to return back to normal.

Figure 8 maps PM^*^ and ENKF^*^ over the Western United States and CA for the months of April for the 2015–2018 period. As anticipated, results illustrate that April 2015 RZSM levels were lower than those in other years. In addition, over the Western United States in general, spatial patterns in ENKF^*^ run for the month of April appear to be more consistent with CHIRPS precipitation anomalies than baseline PM^*^ results lacking SMAP assimilation. Therefore, while SMAP assimilation adds little to time series of spatial averages (see Figure 5), it does appear to improve the accuracy of RZSM^*^ spatial patterns.

## 5. Discussion

In general, both the PM and ENKF RZSM products properly represent the drought events targeted in this paper. However, close comparison between the PM and ENKF results reveal certain key differences (see Figures 3, 6–8). Several anomaly changes are seen in the ENKF product run that are not evident in the PM model run (see boxes in Figure 3). These changes reflect periods of missing precipitation events and, therefore, inadequate PM-only RZSM anomaly response. However, in many cases, the assimilation of SMAP soil Level 2 SM data adequately compensates for missing rainfall and/or misrepresented rainfall events, and ENKF RZSM results demonstrate better *temporal* agreement with baseline CHIRPS data. Furthermore, SMAP properly adjusts the model *spatial* variability and makes it more in line with the spatial variability captured by CHIRPS (see Figures 6–8). The increase in correlation values in Figure 4 is the largest for South Africa followed by NSW. Overall, results demonstrate the value of SMAP and its potential to improve the USDA-FAS crop forecasting system.

The relationship between NDVI and PM and ENKF RZSM estimates are also examined. However, due to lagged nature of the RZMS/NDVI relationship and the tendency for irrigation to de-coupled NDVI and RZSM anomalies in agricultural areas, the vegetation-weather relationship is not always straightforward, which makes it difficult to interpret NDVI behavior relative to the soil moisture and precipitation (Figures 6–8) and estimate the anticipated drought impact on crop growth and yield. Nevertheless, the NDVI anomaly response captured in Figures 3, 5 follows the general pattern evident in the RZSM results. Soil moisture responds rapidly and instantaneously to rainfall and the changes in soil moisture conditions occur almost simultaneously. However, changes in vegetation status is somewhat delayed since plants employ coping mechanisms to deal with water and heat stress. Therefore, crop status will not change in response to an intermittent rainfall event during a period of extended below normal precipitation conditions. Likewise, crops may not recover after prolonged drought and the NDVI response may remain negative during above average precipitation period following a drought. This pattern is observed over drought conditions in NSW between mid-2018 and early 2019. While a small positive spike in rainfall is recorded at the very end of 2018, the NSW NDVI^*^ series remains negative indicating poor vegetation health through the whole period. These dynamics illustrate the difficulty of verifying RZSM dynamics using NDVI alone.

## 6. Summary and Implications

This paper focused on a data assimilation system designed to enhance the USDA-FAS root-zone soil moisture (RZSM) information via the assimilation of SMAP Level 2 surface soil moisture retrievals into the USDA-FAS Palmer model (PM). The resulting PM+SMAP assimilation system is one of the first operational systems to use SMAP data products in a decision support context (Mladenova et al., 2019).

Overall, results demonstrated the benefit of assimilating SMAP and confirmed its potential to improve the USDA-FAS RZSM information generated by the Palmer model. The satellite-based observations were able to enhance the spatial variability of the PM root-zone soil moisture, adjust the model performance and make its response more coherent with the CHIRPS precipitation.

As discussed above, drought is a complex phenomenon. Vegetation growth is directly dependent on the amount of water present in the root-zone; however, RZSM is not the sole factor determining crop health and yield formation. RZSM monitors only one aspect of the drought manifestation (i.e., water deficiency) and as our NDVI analysis demonstrated, exploiting this information to predict future change in crop health may be challenging. The complex plant-weather relationship and the adaptive mechanism plants have to handle environmental stresses complicate direct inter-comparisons between NDIV and SM and precipitation. Our NDVI-based results showed some temporal offset between the soil moisture and NDVI responses to drought; however, this relationship was not immediately apparent and varied between sites. This indicates that the use of root-zone soil moisture dynamics to verify and predict NDVI variability is not straightforward and requires further study. One possible approach would be to consider a more complex methodology, where the precipitation driven variability in RZSM is evaluated relative to air temperature, water levels, snow melted water, wind speed, etc.

SMAP began operational data production in early 2015 and the SMAP data record is still relatively short. This complicates the calculation of interannual anomalies. Despite this complication, the analyses presented here demonstrate that the ENKF data outperform the PM-only run and that SMAP adds additional variability and information, which effectively improves the quality of RZSM information operationally available to the USDA-FAS. However, the short duration of the operational passive-based satellite sensors such as SMAP makes it challenging to set up a stable data assimilation system, compute dependable climatological anomalies and sample statistically significant differences in performance metrics.

One of the main assumptions of data assimilation is that the systematic differences between the model and the satellite observations are accounted for prior to assimilation. This is often achieved using long-term, climatology-based statistical rescaling techniques. Furthermore, while a large number of approaches are applied to detect and track drought, the most commonly used indices are anomaly-based (Keyantash and Dracup, 2002; Mishra and Singh, 2010). Along with SMAP, there have been several other passive-based global systems that have and/or currently provide global soil moisture observations, i.e., the Advanced Microwave Scanning Radiometer (AMSR-E operational between 2002 and 2010 and its successor AMSR2 launched in 2012) and Soil Moisture Ocean Salinity (launched in 2010). Combing these missions within a single data assimilation framework would allow for the generation of a consistent long-term RZSM database for USDA-FAS using the existing satellite-enhanced Palmer model and satellite-based retrievals acquired from AMSR-E, AMSR2, SMOS and SMAP.

RZSM and NDVI are essential parts of the USDA-FAS data repository. As clarified, RZSM provides information on the plant available water, while NDVI provides valuable vegetation related information such as vegetation dynamics, crop health and phenology. Therefore, RZSM and NDVI are both key components of the USDA-FAS's CADRE data management system and play a central role in the agency's crop monitoring and yield forecasting analysis. RZSM and NDVI show a somewhat lagged agreement (in time) and appear to provide complimentary information-since the value of each variable for crop monitoring changes throughout the growing season (Peled et al., 2010; Bolten and Crow, 2012; Han et al., 2014; Mladenova et al., 2017). Better knowledge of the RZSM-NDVI relationship, and proper understating of its temporal variability as a function of crop growth stages, would enhance our ability predict potential change in crop health due to agricultural drought using RZSM information, which, in turn, would aid and improve the yield forecasting. This relationship and its potential to predict change in crop health and the generation of a combine NDVI-RZSM index for drought monitoring and yield forecasting have not been fully explored yet. Filling this gap is a logical next step.

## Data Availability Statement

All data sets analyzed in this study are publicly available. The NASA-USDA soil moisture products generated from the SMAP-enhanced Palmer model can be found here: https://gimms.gsfc.nasa.gov/SMOS/SMAP/. The NASA MODIS NDVI data were acquired using the following link: https://gimms.gsfc.nasa.gov/MODIS/. The CHIPRS precipitation data are distributed by the Climate Hazard Group (http://chg.geog.ucsb.edu/data/index.html).

## Author Contributions

IM has processed the datasets and conducted the analysis presented in the paper. IM have worked on integrating the SMAP satellite observations into the Palmer model and worked with NS on setting up the operational data assimilation system. IM, WC, and JB have worked collaboratively on structuring and writing the paper. WC and JB have helped with reviewing the manuscript and discussing the results. NS has run the Palmer model and helped with initial data processing. CR have provided insides related to the use of the SMAP-enhanced Palmer output by USDA-FAS as well as its value for the agency's crop monitoring activities.

### Conflict of Interest

The authors declare that the research was conducted in the absence of any commercial or financial relationships that could be construed as a potential conflict of interest.
